# The effects of neighborhood socioeconomic status on ADL/IADL among Chinese older adults-neighborhood environments as mediators

**DOI:** 10.3389/fpubh.2023.1202806

**Published:** 2024-01-08

**Authors:** Xiaoshuang Tan, Hong Zhang, Xiaohui Ren

**Affiliations:** ^1^Department of Health Behavior and Social Medicine, West China School of Public Health and West China Fourth Hospital, Sichuan University, Chengdu, China; ^2^Integrated Care Management Center, West China Hospital, Sichuan University, Chengdu, China

**Keywords:** neighborhood SES, activities of daily living, instrumental activities of daily living, neighborhood built environment, neighborhood social environment, mediating effect

## Abstract

**Background:**

There have been few consistencies in the effects and pathways of neighborhood socioeconomic status (SES) on functional limitations. This study aimed to investigate whether neighborhood socioeconomic status influences ADL/IADL in older residents in China through the neighborhood built environment and social environment.

**Methods:**

Activities of daily living/IADL were assessed in a sample of 5,887 Chinese individuals aged 60 or older, utilizing data obtained from the 2011 China Health and Retirement Longitudinal Study (CHARLS 2011). Neighborhood SES was measured by the neighborhood per-capita net income. Neighborhood built environment was measured by the security resources, motion resources, living resources, service resources for older adults, and medical resources of neighborhood. Neighborhood social environment was measured by the organizations, unemployment subsidies, minimum living allowance, subsidies to persons older than 65, and pensions to persons older than 80 of the neighborhood. The two-level logistical regression model and multilevel structural equation model (MSEM) were used.

**Results:**

The rate of ADL/IADL loss among Chinese older adults aged 60 and above in 2011 were 32.17 and 36.87%, respectively. Neighborhood SES was significantly associated with ADL/IADL in older adults. Compared with the respondents living in communities with lower SES, those living in communities with higher SES possessed better ADL (*β* = −0.33, *p* < 0.05) and IADL (*β* = −0.36, *p* < 0.05) status. The path of neighborhood socioeconomic status on ADL was completely mediated by the neighborhood built environment (*β* = −0.110, *p* < 0.05) and neighborhood social environment (*β* = −0.091, *p* < 0.05). Additionally, the effect of neighborhood socioeconomic status on IADL was fully mediated by the neighborhood built environment (*β* = −0.082, *p* < 0.05) and neighborhood social environment (*β* = −0.077, *p* < 0.05).

**Conclusion:**

Neighborhood SES was significantly correlated with ADL/IADL through the neighborhood environment. Improving the ADL/IADL status of older adults residing in low socioeconomic neighborhoods requires enhancing the built and social environment by provisioning additional neighborhood resources.

## 1 Introduction

Population aging is a major global trend as life expectancy rises, driven by advances in healthcare, medical conditions, improved access to education, and declining fertility rates (1). According to the World Social Report 2023, the worldwide population of individuals aged 65 and above reached 761 million in 2021, with projections suggesting a rise to 1.6 billion by 2050 (2). There is an enduring, mostly irreversible pattern toward an aging demographic in numerous countries. This group is considered high-risk for various diseases, and the advanced age and multiple medical conditions in older adults can lead to disability, resulting in a loss of self-care, decreased quality of life and life expectancy, and an increased risk of depression and suicide (3, 4). This, in turn, leads to increased healthcare needs and prolonged care requirements for older adults, putting a strain on healthcare and social welfare systems.

Disability is commonly measured by activities of daily living (ADLs) and instrumental activities of daily living (IADLs), which are important indicators of older adults’ ability to care for themselves, their quality of life, and their daily health care needs (5, 6). In prior research, ADL is frequently combined with IADL, and the two concepts are interrelated, with ADL being the more severe form (7) and IADL being the most potent predictor of ADL (8). The overall outlook for functional limitations in older adults is disheartening, with around 29% of individuals aged 65 and older who were registered in United States Medicare programs experiencing at least one ADL loss, and roughly 12% of older adults having trouble with one or more IADLs without any ADL limitations (9). A multivariate study indicated that 24.8% of individuals over the age of 70 in Germany had ADL loss, while 45.8% reported IADL limitations (8). In Brazil, 32.7% of the participants aged 60 and over were unable to perform at least one IADL and/or ADL, and 18.1% of the population suffered from ADL disabilities (10). In China, the number of older adults with ADL loss was projected to increase from 8.4 million in 2010 to 37 million in 2050, resulting in a high burden of care and medical costs for individuals, families and society (11).

Neighborhood socioeconomic status (SES), which encompasses educational, occupational, and economic resources (12), reflects inequalities in macro-resources and neighborhood environmental resources. As one of the neighborhood characteristics, SES serves as a commonly used indicator to assess environmental quality (13) in addition to being closely related to the health of the older adults (14, 15). In the study of neighborhood SES and ADL loss among older adults, Philibert et al. (16) found that there was a positive correlation between neighborhood SES and the non-loss rate of ADL/IADL among older adults, the higher the neighborhood SES, the higher the non-loss rate of ADL/IADL. Freedman et al. discovered that men residing in neighborhoods with lower socioeconomic status had a higher risk of ADL/IADL limitation, whereas no such relationship was found for women (17). However, Glymour et al. (18) argued that there was no statistically significant association between low neighborhood SES and ADL loss. Therefore, current studies have not reached a consistent conclusion regarding the connection between neighborhood SES and functional limitations in older adults.

In addition, few studies have investigated the exact mechanism by which neighborhood SES affects ADL/IADL. According to the social-ecological model, we speculated that the community environment may be a potential influencing factor that was closely associated with the socioeconomic status (SES) of the community (19). As Chong et al. found, communities with higher socioeconomic status have better infrastructure, such as educational facilities, recreational facilities, and public transportation, and residents have access to more employment opportunities, a wider range of material resources, and social benefits (20). On the other hand, communities with higher socioeconomic status are less likely to be exposed to violent and chaotic environments and have access to more economic resources (21), thus helping to build a variety of activity organizations to enrich the lives of older adults and create a harmonious social atmosphere. Generally, the built environment and the social environment are two domains of the neighborhood environment (22). Specifically, the neighborhood built environment refers to the physical aspects surrounding residential homes that can be altered by policies and behaviors, such as public infrastructure, road networks, and public spaces (23). The health-related neighborhood social environment is summarized to comprise neighborhood socioeconomic status, social capital, social services, and social security, etc. (22, 24)

Among the existing studies on neighborhood built environment and functional disabilities in older adults, Balfour’s study showed that inadequate lighting and excessive noise were associated with functional limitations (25). Poor street conditions in the community, including the absence of crosswalks and uneven surfaces, exacerbate the likelihood of older adults experiencing ADL loss, while also worsening the condition of those who already exhibit impaired functional status (26). Gobbens’ study indicated that neighborhoods with increased safety and reduced crime rates had a lower occurrence of ADL/IADL loss among older adults, while street conditions (streetscapes, pathways, and lighting) were only associated with IADL (27). In terms of neighborhood esthetics, older adults residing in neighborhoods with poor environmental cleanliness and high levels of disorder, such as intentional graffiti or vandalism, experienced a greater incidence of ADL/IADL loss (28), whereas neighborhood cleanliness was only associated with IADL in Qin’s study (7). In contrast, exposure to green spaces surrounding residential areas enhances physical functioning, diminishes losses in ADL/IADL, and mitigates the burden of long-term care for older adults (29). However, in Peng’s study, exposure to green spaces surrounding residential areas was only associated with ADL (30).

Concerning the social environment in neighborhoods, Cao found that social capital, as measured by neighborhood trust involvement, had a significant positive effect on ADL among older adults in both urban and rural communities in Indonesia (31). After distinguishing social participation at the individual and neighborhood levels, Oshio found that high levels of neighborhood social participation can reduce the likelihood of ADL loss among retired older adults (32). Similarly, older adults with lower levels of social capital had greater odds of ADL/IADL loss, particularly in terms of perceived neighborhood cohesion (33). And in a study of neighborhood cohesion through an ecological lens and longitudinal survey, Qin found that neighborhood social cohesion protects the ability of community-dwelling older adults to engage in self-care and domestic activities and may reduce the odds of ADL/IADL loss in older adults (7). Based on the aforementioned evidence, do neighborhood built environment and neighborhood social environment serve as mediating variables in the pathway by which community SES affects ADL/IADL?

As the world’s largest country with an aging population, China’s older adults over the age of 65 have reached 190 million (13.5%) in 2020 (31). Due to the significant effects of aging, the inconsistent relationship and unclear mechanisms between neighborhood SES and ADL/IADL, this study utilized data from CHARLS 2011 to investigate how neighborhood SES impacts ADL/IADL in older adults, as well as whether it affects functional limitations through the neighborhood’s built or social environment. It may serve as a guide for enhancing older adults’ self-care abilities and overall quality of life by improving the surrounding community environment, specifically in areas with lower socioeconomic status.

## 2 Materials and methods

### 2.1 Data

China Health and Retirement Longitudinal Study is a national longitudinal survey of Chinese residents aged 45 and above, aimed at analyzing the trend of population aging and promoting interdisciplinary research on aging. The CHARLS sample was collected via multistage stratified probability proportional to size (PPS) sampling. The baseline survey, conducted in 2011, covered 28 provinces, 150 counties or districts, 450 communities, and 10,257 households, with 17,706 participants aged 45 years and older. In addition to individual data, data on neighborhood characteristics were also available. The neighborhood data were obtained from local officials, draftsmen, and interviewers’ observations, including aspects such as social, economic, policy, and other community-related factors. It should be noted that CHARLS only collected neighborhood data in 2011, thus restricting our analytic sample to individuals over 60 years old who were interviewed in 2011. After excluding respondents under the age of 60 and communities with fewer than five samples (34), the study was conducted on a final sample size of 5,887 participants from 444 communities. It comprised 3,694 respondents from 237 rural communities and 2,193 respondents from 207 urban communities.

### 2.2 Measurement

#### 2.2.1 Dependent variable

Dependent variables were Activities of Daily Living (ADL) and Instrumental Activity of Daily Living (IADL). ADL was assessed using Katz’s Functional Index of ADL (30), consisting of six items that assess an individual’s ability to perform basic self-care activities such as bathing, eating, dressing, getting on and off the bed, using the toilet, and defecating. Each item was assigned to a corresponding question. IADL was typically assessed using six items, which encompassed cooking, household tasks, medication management, phone communication, shopping, and financial management (6). However, as CHARLS 2011 did not examine the task of making phone calls, our research on IADL only encompasses five components. These components were assigned to five questions, each containing four responses: “have any difficulty,” “have difficulty but can still do it,” “have difficulty and need help,” and “cannot do it.” If the participants completed six items with “have any difficulty,” we defined them as “ADL/IADL without loss” (=0). Conversely, if the respondents finished any items without “have any difficulty,” we categorized them as “ADL/IADL loss” (= 1).

#### 2.2.2 Independent variables

The study used the socioeconomic status (SES) of the neighborhood as an independent variable.

The per-capita net income and literacy index of neighborhood were used to measure neighborhood socioeconomic status (SES). The question on the per-capita net income of neighborhood was: “What was the per-capita net income of this village/community in 2010?” The higher a neighborhood’s per-capita net income, the higher its socioeconomic status. There were six questions to investigate neighborhood literacy level in CHARLS 2011 community questionnaire: (a) What percentage of the adult population is illiterate/semi-illiterate in your village/community?; (b) What percentage of the adult population has only completed primary school in your village/community?; (c) What percentage of the adult population has completed only up to junior middle school in your village/community?; (d) What percentage of the adult population has completed only up to senior high school in your village/community?; (e) What percentage of the adult population has completed only up to college in your village/community?; and (f) What percentage of the adult population has completed only up to graduate school in your village/community? Principal component analysis was used to construct the community literacy index, and the formula was as follows:


(1) $$\begin{array}{l} neighborhood\;literacy\;index=0.04a+0.17b+0.09c\\ \;\;\;\;\;\;\;\;\;\;\;\;\;\;\;\;\;\;\;\;\;\;\;\;\;\;\;\;\;\;\;\;\;\;\;\;\;\;\;\;\;\;\;\;\;\;\;\;\;\;\;\;\;\;\;\;\;\;\;\;\;\;\;\;\;\;\;\;\;\;\;\;\;\;\;+0.15d+0.24e+0.32f \end{array}$$

The range of neighborhood per-capita net income was from 0 to 50,415 RMB. And the range of neighborhood literacy index was from 1.2 to 4.3. The higher the value of the indicator, the higher the level of the indicator. However, since the per-capita net income and the neighborhood literacy index were highly skewed distribution variables, the lower quartile (*P_25_*) and the upper quartile (*P_75_*) were used to divide them into three grades: “high” (=3), “middle” (=2), and “low” (=1). The *P_25_* and *P_25_* of neighborhood per-capita net income were 1,500 and 5,400 RMB, respectively. The *P_25_* and *P_25_* of the neighborhood literacy index were 1.96 and 2.44, respectively.

#### 2.2.3 Mediator variables

We utilized the security resources, motion resources, living resources, service resources for older adults, and medical resources of the community to measure neighborhood built environment. Additionally, we used the organizations, unemployment subsidies, minimum living allowance, subsidy to persons older than 65, and pension to persons older than 80 of the community to measure neighborhood social environment. These neighborhood characteristics have been shown to be associated with older adults’ mental health, such as cognitive function (35), self-rated health (36), and physical health such as cardiovascular disease (37).

In terms of the usage and venues of different facilities in surveyed communities, neighborhood security resources were measured by “whether the community has police stations or police room” (yes = 1, no = 0); Neighborhood motion resources were measured by “whether the community has one of exercise facilities (such as basketball court, swimming pool, outside exercising facilities, table tennis, room for card games and chess games, room for Ping Pong)” (yes = 1, no = 0); Neighborhood living resources were measured by “whether the community has one of living facilities (such as theaters, post offices, banks, farmers’ markets, supermarkets, and other entertainment facilities)” (yes = 1, no = 0); Neighborhood service resources for older adults were measured by “whether the community has one of service facilities for older adults (such as nursing homes, activity center for older adults)” (yes = 1, no = 0). Neighborhood questionnaires covering eight types of medical facilities (such as general hospitals, specialized hospitals, Chinese traditional medicine hospitals, nearby pharmacy stores, community health care centers, community health care medical posts, township health clinics and hospitals, and village medical posts), we defined the absence of any type of medical facility as “no community medical resource” (=0); we defined the presence of at least one type of medical facility as “have community medical resource” (=1).

Among neighborhood social environment, neighborhood unemployment subsidies were measured by “Does your village/community have unemployment subsidies?” (yes = 1, no = 0); Neighborhood minimum living security fund was measured by “Does your village/community have minimum living allowance?” (yes = 1, no = 0); Neighborhood old subsidy was measured by “Does your village/community issue pension older than 65?” (yes = 1, no = 0) and “Does your village/community issue pension older than 80?” (yes = 1, no = 0); Neighborhood organization was measured by “Does your village/community have following type of facilities: associations for calligraphy and painting, dancing teams or other exercise organizations, organizations for helping the older adults and the handicapped, older adults’ associations” (yes = 1, no = 0).

#### 2.2.4 Control variables

The control variables in the study included age, gender, marital status, education level, household per-capita consumption expenditure, and the number of chronic diseases the respondents have. Age was measured in years. Gender was coded as a binary variable (female = 0, male = 1). And marital status, residence were also coded as a binary variable (other = 0, married = 1; rural = 0, urban = 1). Education level was coded into four categories (illiteracy = 1, primary = 2, middle school = 3, high school and above = 4). Household per-capita consumption expenditure was calculated by dividing total household consumption expenditure by the number of family members. Furthermore, because household per-capita consumption expenditure had a highly skewed distribution, it was further divided into three levels (low = 1, middle = 2, and high = 3) by lower (*P_25_ = 1,000 RMB*) and higher (*P_75_ = 6,500 RMB*). The list of chronic diseases includes hypertension, dyslipidemia, diabetes/high blood sugar, cancer/malignant tumors, chronic lung diseases, liver disease, heart attack, stroke, kidney disease, stomach/other digestive disease, emotional, nervous/psychiatric problems, memory-related diseases, arthritis/ rheumatism and asthma. In this study, the total number of these 14 types of diseases was used as an indicator of chronic disease.

**Figure 1:**
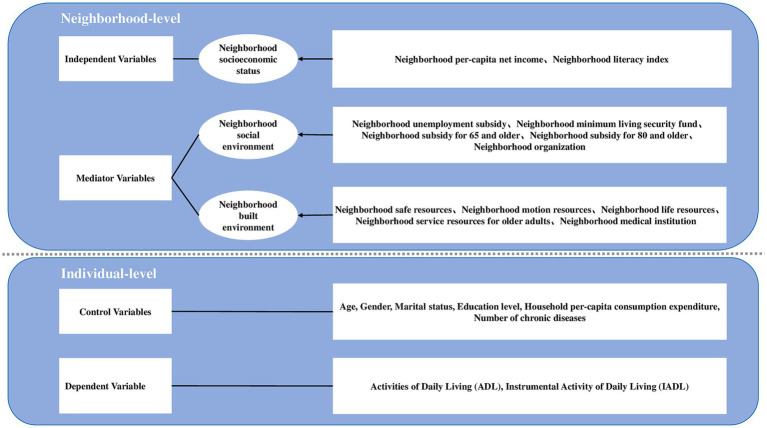
Conceptual framework.

The entire conceptual framework of the research was illustrated in Figure 1.

### 2.3 Data analysis

First, descriptive statistics (prevalence, means, and variances) were employed to examine the distributions and properties of the dependent, independent, mediator, and control variables. Next, independent samples *t*-tests and chi-squared tests were used to test the bivariate association between the dependent and independent, mediator and control variables (continuous and categorical, respectively). Third, given that the data had two levels (community & individual) and the dependent variable was a dichotomous variable, the two-level logistic regression model was applied (38), adjusting for control variables (such as age, education level, and others) was applied to test whether the data were suitable for multilevel statistical analysis and to assess the impact of independent variables on dependent variables. The study filtered variables that were statistically significant in the bivariate association into the two-level logistic regression model. During the statistical analysis, taking into account the aggregation of neighborhood data, we estimated the intraclass correlation coefficient (ICC) (39) for the two-level logistic regression model. The ICC has two main interpretations: firstly, it assesses the percentage of variance in the outcome variable that can be attributed to different aggregation levels, and secondly, it determines the extent to which individual-level observations are correlated within each aggregation level. ICC > 0 indicates the statistical significance of multilevel analysis (40).

Fourth, using the outlined steps, a multilevel structural equation model (MSEM) was utilized to establish the latent variables of neighborhood built environment and neighborhood social environment, and test whether neighborhood environment mediates the pathway by which neighborhood socioeconomic status influences ADLs/IADLs of older adults. MSEM is a general framework that combines the structural equation model (SEM) and multilevel modeling simultaneously. MSEM was conducted in two steps, the first of which used confirmatory factor analysis to establish a measurement of neighborhood built environment and neighborhood social environment. Only observation variables with factor loadings greater than 0.6 were eligible for inclusion in the measurement model. In the second step, a multilevel structural model was conducted by entering dependent variables, neighborhood socioeconomic status, and control variables. Fit indices were used to determine how well the model fit the data. The fit indices and cutoff points were shown below: Chi-square test (chi-square/df less than 3), comparative fit index (CFI; estimates more than 0.9), root mean square error of approximation (RMSEA; estimates less than 0.08), and standardized root mean square residual of within and between groups (SRMR-W and SRMR-B; estimates less than 0.08) (41, 42).

The study implemented a two-level logistic regression model utilizing Stata 15.1. Additionally, a multilevel structural equation model was carried out utilizing Mplus 7.0. The significance level for this study was set at *α* = 0.05.

## 3 Results

### 3.1 Descriptive statistics of the respondent’s variables

Among 5,887 respondents, 32.17 and 36.87% experienced ADL/IADL impairment, respectively. Merely 19.96% of the sample reported an absence of the 14 physician-diagnosed chronic illnesses. The average age of the respondents was 69.18 ± 7.27 years old, while over half had an education level of illiteracy (60.85%). A majority of the respondents were married (75.66%). More than half of the respondents were female (54.49%). More than half of the population (62.75%) lived in rural areas. In addition, 27.45% of the respondents reported a low per-capita household consumption expenditure (Table 1).

**Table 1:** Characteristics of respondents’ sociodemographic characteristics and health status.

Variable	Value
ADL *n* (%)	
ADL loss	1,894 (32.17)
ADL without loss	3,993 (67.83)
IADL *n* (%)	
IADL loss	2,165 (36.87)
IADL without loss	3,707 (63.13)
Number of chronic diseases $\bar{x}$ (SD)	1.84 (1.52)
Age $\bar{x}$ (SD)	69.18 (7.27)
Literacy *n* (%)	
Illiteracy	3,582 (60.85)
Primary	1,380 (23.44)
Middle school	591 (10.04)
High school and above	335 (5.67)
Marital status *n* (%)	
Married	4,454 (75.66)
Other marital status	1,433 (24.34)
Gender *n* (%)	
Male	2,677 (45.51)
Female	3,205 (54.49)
Residence *n* (%)	
Urban	2,193 (37.25)
Rural	3,694 (62.75)
Household per-capita consumption expenditure *n* (%)	
Low	1,591 (27.45)
Middle	2,772 (47.82)
High	1,434 (24.74)

ADL, Activities of daily living.

### 3.2 Characteristics of the communities

As shown in Table 2, 24.64% of the 444 communities had low per-capita net income. 27.45% of the communities had a low-level literacy index. Less than half of the communities owned safe resources (48.65%). More than half of communities had motion resources such as basketball courts, swimming pools, and others (65.24%). The majority was owned by life resources such as farmers’ markets, convenience stores, and others (94.14%). 46.40% of communities had service resources for older adults such as activity centers for the older adults and so on. 19.41% of communities had no medical institutions. Only 14.93% of communities had unemployment subsidies. Over half of communities had a minimum living security fund (82.39%). The minority communities issued subsidies to persons older than 65 years (22.78%). Also, 30.68% of communities issued subsidies to persons older than 80 years (30.68%). 56.66% of communities formed all kinds of neighborhood organizations, such as organizations for helping the older adults and handicapped, dancing teams or other exercise organizations, and so on.

**Table 2:** Characteristics of neighborhood variables.

Variable	*N* (%)
Neighborhood per-capita net income	
Low	102 (24.64)
Middle	192 (46.38)
High	120 (28.99)
Neighborhood literacy index	
Low	115 (27.45)
Middle	191 (45.58)
High	113 (26.97)
Neighborhood safe resources	
Yes	216 (48.65)
No	228 (51.35)
Neighborhood motion resources	
Yes	289 (65.24)
No	154 (34.76)
Neighborhood life resources	
Yes	418 (94.14)
No	26 (5.85)
Neighborhood service resources for older adults	
Yes	206 (46.40)
No	238 (53.60)
Neighborhood medical institutions	
Yes	357 (80.59)
No	86 (19.41)
Neighborhood unemployment subsidies	
Yes	66 (14.93)
No	376 (85.07)
Neighborhood minimum living security fund	
Yes	365 (82.39)
No	78 (17.61)
Neighborhood subsidy for 65 and older	
Yes	100 (22.78)
No	339 (77.22)
Neighborhood subsidy for 80 and older	
Yes	135 (30.68)
No	305 (69.32)
Neighborhood organizations	
Yes	251 (56.66)
No	192 (43.34)

#### 3.2.1 Association between ADL and individual/neighborhood characteristics

As shown in Table 3, there was a significant (*p* < 0.05) difference in the distribution of ADL based on the number of chronic diseases, age, literacy, marital status, gender, residence, and household per-capita consumption expenditure. Among the neighborhood-level variables, the differential distribution of ADL on neighborhood per-capita net income, neighborhood safe resources, neighborhood motion resources, neighborhood service resources for older adults, neighborhood unemployment subsidies, and neighborhood organization were significant (*p* < 0.05). However, the ADL differential distribution on neighborhood literacy index, neighborhood life resources, neighborhood medical institutions, neighborhood minimum living security funds, neighborhood subsidies for 65 and older, and neighborhood subsidies for 80 and older were not statistically significant (*p* > 0.05).

**Table 3:** Bivariate association between ADL and individual- and neighborhood-level variables.

Variable	ADL		χ^2^/*t* value	*p*
	ADL loss [*n* (%)]	ADL without loss [*n* (%)]		
Individual-level variables				
Number of chronic diseases [ $\bar{x}$ (SD)]	2.26 (0.04)	1.64 (0.02)	−14.928	<0.001
Age [ $\bar{x}$ (SD)]	70.67 (0.17)	68.47 (0.11)	−10.925	<0.001
Literacy			77.317	<0.001
Illiteracy	1,298 (36.24)	2,284 (63.76)		
Primary	380 (27.54)	1,000 (72.46)		
Middle school	151 (25.55)	440 (74.45)		
High school and above	65 (19.46)	269 (80.54)		
Marital status			20.104	<0.001
Married	1,364 (30.62)	3,090 (69.38)		
Other marital status	530 (36.99)	903 (63.01)		
Gender			5.982	0.014
Male	817 (30.52)	1,860 (69.48)		
Female	1,074 (33.51)	2,131 (66.49)		
Urban–rural			32.340	<0.001
Urban	607 (27.68)	1,586 (72.32)		
Rural	1,287 (34.84)	2,407 (65.16)		
Household per-capita consumption expenditure			7.996	0.018
Low	478 (30.04)	1,113 (69.96)		
Middle	940 (33.91)	1,832 (66.09)		
High	445 (30.03)	989 (68.97)		
Neighborhood-level variables				
Neighborhood per-capita net income			25.221	<0.001
Low	501 (34.50)	951 (65.50)		
Middle	924 (34.00)	1,794 (66.00)		
High	372 (26.90)	1,011 (73.10)		
Neighborhood literacy index			5.541	0.063
Low	503 (31.86)	1,076 (68.14)		
Middle	894 (33.81)	1,750 (66.19)		
High	424 (30.26)	977 (69.74)		
Neighborhood safe resources			67.038	<0.001
Yes	637 (26.22)	1,792 (73.78)		
No	1,257 (36.35)	2,201 (63.65)		
Neighborhood motion resources			51.807	<0.001
Yes	972 (28.43)	2,447 (71.57)		
No	918 (37.32)	1,542 (62.68)		
Neighborhood life resources			0.256	0.613
Yes	1,765 (32.09)	3,735 (67.91)		
No	129 (33.33)	258 (66.67)		
Neighborhood service resources for older adults			46.139	<0.001
Yes	629 (27.05)	1,696 (72.95)		
No	1,265 (35.51)	2,297 (64.49)		
Neighborhood medical institution			0.019	0.890
Yes	1,508 (32.22)	3,173 (67.78)		
No	386 (32.01)	820 (67.99)		
Neighborhood unemployment subsidy			39.538	<0.001
Yes	138 (21.30)	510 (78.70)		
No	1,750 (33.53)	3,469 (66.47)		
Neighborhood minimum living security fund			0.413	0.521
Yes	1,570 (32.38)	3,278 (67.62)		
No	322 (31.35)	705 (68.65)		
Neighborhood subsidy for 65 and older			1.000	0.317
Yes	388 (31.06)	861 (68.94)		
No	1,494 (32.56)	3,095 (67.44)		
Neighborhood subsidy for 80 and older			0.707	0.401
Yes	534 (31.41)	1,166 (68.59)		
No	1,346 (32.54)	2,790 (67.46)		
Neighborhood organization			46.305	<0.001
Yes	786 (27.85)	2,044 (72.15)		
No	1,101 (36.15)	1,945 (63.85)		

#### 3.2.2 Association between IADL and individual/neighborhood characteristics

As displayed in Table 4, a notable distinction exists (*p* < 0.05) in the allocation of IADL depending on the number of chronic diseases, age, literacy, marital status, gender, residence, and household per-capita consumption expenditure. Among the variables at the neighborhood level, the distribution of IADL based on per-capita net income, literacy index, safe resources, motion resources, service resources for older adults, unemployment subsidies, subsidy for 80 and older, and organization were found to be significantly significant (*p* < 0.05). However, the IADL differential distribution on neighborhood life resources, neighborhood medical institutions, neighborhood minimum living security funds, and neighborhood subsidies for 65 and older were not statistically significant (*p* > 0.05).

**Table 4:** Bivariate association between IADL and individual- and neighborhood-level variables.

Variable	IADL		χ^2^/*t* value	*p*
	IADL loss [*n* (%)]	IADL without loss [*n* (%)]		
Individual-level variables				
Number of chronic diseases	2.16 (0.04)	1.65 (0.02)	−12.79	<0.001
Age	71.17 (0.17)	68.00 (0.11)	−16.51	<0.001
Literacy			189.41	<0.001
Illiteracy	1,559 (43.68)	2,010 (56.32)		
Primary	389 (28.23)	989 (71.77)		
Middle school	150 (25.38)	441 (74.62)		
High school and above	67 (20.06)	267 (79.94)		
Marry status			64.21	<0.001
Married	1,513 (34.02)	2,935 (65.98)		
Other marital status	652 (45.79)	772 (54.21)		
Gender			40.10	<0.001
Male	1,295 (40.53)	1,900 (59.47)		
Female	869 (32.52)	1,803 (67.48)		
Residence			27.86	<0.001
Urban	712 (32.56)	1,475 (67.44)		
Rural	1,453 (39.43)	2,232 (60.57)		
Household per-capita consumption expenditure			17.28	<0.001
Low	522 (32.87)	1,066 (67.13)		
Middle	1,083 (39.18)	1,681 (60.82)		
High	525 (36.69)	906 (63.31)		
Neighborhood-level variables				
Neighborhood per-capita net income			41.40	<0.001
Low	558 (38.48)	892 (61.52)		
Middle	1,081 (39.87)	1.630 (60.13)		
High	412 (29.83)	969 (70.17)		
Neighborhood literacy index			6.73	0.035
Low	601 (38.16)	974 (61.84)		
Middle	994 (37.72)	1,641 (62.28)		
High	477 (34.05)	924 (65.95)		
Neighborhood safe resources			45.20	<0.001
Yes	771 (31.82)	1,652 (68.18)		
No	1,394 (40.42)	2,055 (59.58)		
Neighborhood motion resources			71.10	<0.001
Yes	1,103 (32.33)	2,309 (67.67)		
No	1,057 (43.09)	1.396 (56.91)		
Neighborhood life resources			0.53	0.466
Yes	2,029 (36.99)	3,456 (63.01)		
No	136 (35.14)	251 (64.86)		
Neighborhood service resources for older adults			50.74	<0.001
Yes	727 (31.32)	1,594 (68.68)		
No	1,438 (40.50)	2,113 (59.50)		
Neighborhood medical institution			0.13	0.715
Yes	1,716 (36.75)	2,953 (63.25)		
No	449 (37.32)	754 (62.68)		
Neighborhood unemployment subsidy			29.82	<0.001
Yes	175 (27.05)	472 (72.95)		
No	1,980 (38.03)	3,227 (61.97)		
Neighborhood minimum living security fund			1.53	0.22
Yes	1,766 (36.50)	3,073 (63.50)		
No	394 (38.55)	628 (61.45)		
Neighborhood subsidy for 65 and older			0.93	0.334
Yes	475 (38.12)	771 (61.88)		
No	1,677 (36.63)	2,901 (63.37)		
Neighborhood subsidy for 80 and older			9.94	0.002
Yes	678 (40.02)	1,016 (59.98)		
No	1,471 (35.63)	2,675 (64.37)		
Neighborhood organization			77.17	<0.001
Yes	879 (31.09)	1,948 (68.91)		
No	1,281 (42.17)	1,757 (57.83)		

#### 3.2.3 The result of two-level logistical regression model on ADL loss

Table 5 examined whether the data were suitable for multilevel statistical analysis and the effects of neighborhood-level variables on ADL loss. The null model showed that the ICC was greater than 0 (ICC = 15.47%), meaning that 15.47% of the variation in ADL came from the neighborhood environment, which proved that the data were suitable for the two-level logistic regression model.

**Table 5:** Two-level logistic regression model for respondents’ ADL in China.

Model predictors (reference)	Null model	Model 1
	*β*. (S.E.)	*β*. (S.E.)
Control variables		
Number of chronic diseases		0.31(0.02)^***^
Age		0.05(0.01)^***^
Literacy (Illiteracy)		
Primary		−0.32(0.09)^***^
Middle school		−0.34(0.13)^***^
High school and above		−0.78(0.19)^***^
Marital status (Other marital status)		
Married		−0.02(0.08)
Gender (Female)		
Male		−0.15(0.07)^**^
Residence (Rural)		
Urban		−0.32(0.12)^***^
Per-capita household consumption expenditure (Low)		
Middle		0.24(0.08)^***^
High		0.21(0.10)^**^
Neighborhood-level variables		
Neighborhood per-capita net income (Low)		
Middle		−0.04(0.13)
High		−0.33(0.16)^**^
Neighborhood literacy index (Low)		
Middle		0.02(0.13)
High		−0.07(0.15)
Constant	−0.91(0.05)^***^	−5.07(0.42)^***^
Neighborhood-level variance	0.60(0.08)	0.65(0.09)
Log likelihood	−3573.40	−2977.63
ICC	15.47%	

^*^p < 0.10, ^**^p < 0.05, and ^***^p < 0.01.

As shown in Table 5, Model 1 included control variables and independent variables, which indicated that neighborhood per-capita net income was significantly correlated with ADL loss. Compared to the respondents living in communities with low per-capita net income, those living in communities with low per-capita net income possessed higher rates of ADL loss (*β* = −0.33, *p* < 0.05). In addition, the number of chronic diseases was positively associated with ADL loss, and the more types of chronic diseases respondents developed, the worse their ADL status was (*β* = 0.31, *p* < 0.05). The risk of ADL loss also increased as respondents got older (*β* = 0.05, *p* < 0.05). Compared to individuals with a higher level of education, those who were illiterate have a poorer status ADL status. Females were also more likely to have ADL loss (*β* = −0.15, *p* < 0.05). Respondents living in rural areas had more rates of ADL loss (*β* = −0.32, *p* < 0.05). Furthermore, respondents with high household per-capita consumption expenditure were more prone to experiencing ADL loss. Marital status and community literacy index, on the other hand, were not significant covariates with ADL (*p* > 0.05).

#### 3.2.4 The result of two-level logistical regression model on IADL

Table 6 assessed the appropriateness of utilizing multilevel statistical analysis on the data and evaluated the effects of neighborhood-level variables on IADL. The null model demonstrated an ICC greater than 0 (ICC = 11.63%), indicating that 11.63% of the variation in IADL was attributable to the neighborhood environment. Therefore, the data were suitable for a two-level logistic regression model.

**Table 6:** Two-level logistic regression model for respondents’ IADL in China.

Model predictors (reference)	Null model	Model 1
	*β*. (S.E.)	*β*. (S.E.)
Control variables		
Number of chronic diseases		0.27 (0.02)^***^
Age		0.07 (0.01)^***^
Literacy (Illiteracy)		
Primary		−0.49 (0.09)^***^
Middle school		−0.62 (0.13)^***^
High school and above		−1.18 (0.19)^***^
Marry status (Other marital status)		
Married		−0.10 (0.08)
Gender (Female)		
Male		−0.24 (0.07)^***^
Residence (Rural)		
Urban		−0.22 (0.11)^**^
Per-capita household consumption expenditure (Low)		
Middle		0.45 (0.08)^***^
High		0.47 (0.10)^***^
Neighborhood-level variables		
Neighborhood per-capita net income (Low)		
Middle		0.16 (0.12)
High		−0.36 (0.14)^**^
Neighborhood literacy index (Low)		
Middle		−0.14 (0.12)
High		−0.12 (0.14)
Constant	−0.65 (0.04)^***^	−5.98 (0.41)^***^
Neighborhood-level variance	0.43 (0.06)	0.48 (0.07)
Log likelihood	−3773.78	−3050.29
ICC	11.63%	-

^*^p < 0.10, ^**^p < 0.05, and ^***^p < 0.01.

Table 6 shows that neighborhood per-capita net income had a significant correlation with IADL loss in Model 1, which included both control and independent variables. Those living in communities with high per-capita net income had better IADL status compared to those residing in areas with low per-capita net income (*β* = −0.36, *p* < 0.05). Additionally, the number of chronic diseases was positively associated with IADL loss, and the more types of chronic diseases respondents developed, the worse their IADL status was (*β* = 0.27, *p* < 0.05). The risk of IADL loss also increased as respondents got older (*β* = 0.07, *p* < 0.05). Respondents with primary, middle, and high school education and above had better IADL status in comparison to illiterate respondents. Females had a greater likelihood of experiencing IADL loss (*β* = −0.24, *p* < 0.05). Respondents living in urban areas had better IADL status (*β* = −0.22, *p* < 0.05). Furthermore, respondents with high household per-capita consumption expenditure owned worse IADL. However, marital status and community literacy index did not have a significant association with IADL loss (*p* > 0.05).

### 3.3 Multilevel structural equation model

#### 3.3.1 Measurement model of neighborhood built environment and neighborhood social environment

Before conducting the multilevel structural equation model, we applied a measurement model to establish two latent variables (neighborhood built environment and neighborhood social environment). Table 7 showed the model fit index and the standardized estimates of the factor loadings in the measurement model. The fit index estimates indicated that the model fit was good: χ^2^/df = 62.19, *p* < 0.001, CFI = 0.977, TLI = 0.944, and RMSEA = 0.102. The standardized estimates of factor loadings ranged from 0.715 to 0.826 for the latent variable of neighborhood built environment. Because of the factor loading of neighborhood subsidy for 80 and older was less than 0.5 (39), the neighborhood social environment was measured by neighborhood unemployment subsidies and neighborhood organization. The standardized estimates of factor loadings ranged from 0.563 to 0.853 for the latent variable of neighborhood social environment. Also, the study used only neighborhood per-capita net income to measure neighborhood SES since the factor loading of the neighborhood literacy index was less than 0.5.

**Table 7:** Factor loadings of measurement model.

Latent variable	Observational variable	Factor loading	S.E.	*p*
Neighborhood built environment	Neighborhood safe resources	0.715	0.013	<0.001
	Neighborhood motion resources	0.760	0.013	<0.001
	Neighborhood service resources for older adults	0.826	0.011	<0.001
Neighborhood social environment	Neighborhood unemployment subsidies	0.563	0.023	<0.001
	Neighborhood organization	0.853	0.017	<0.001

##### 3.3.1.1 Result of multilevel structural equation model on ADL

**Figure 2:**
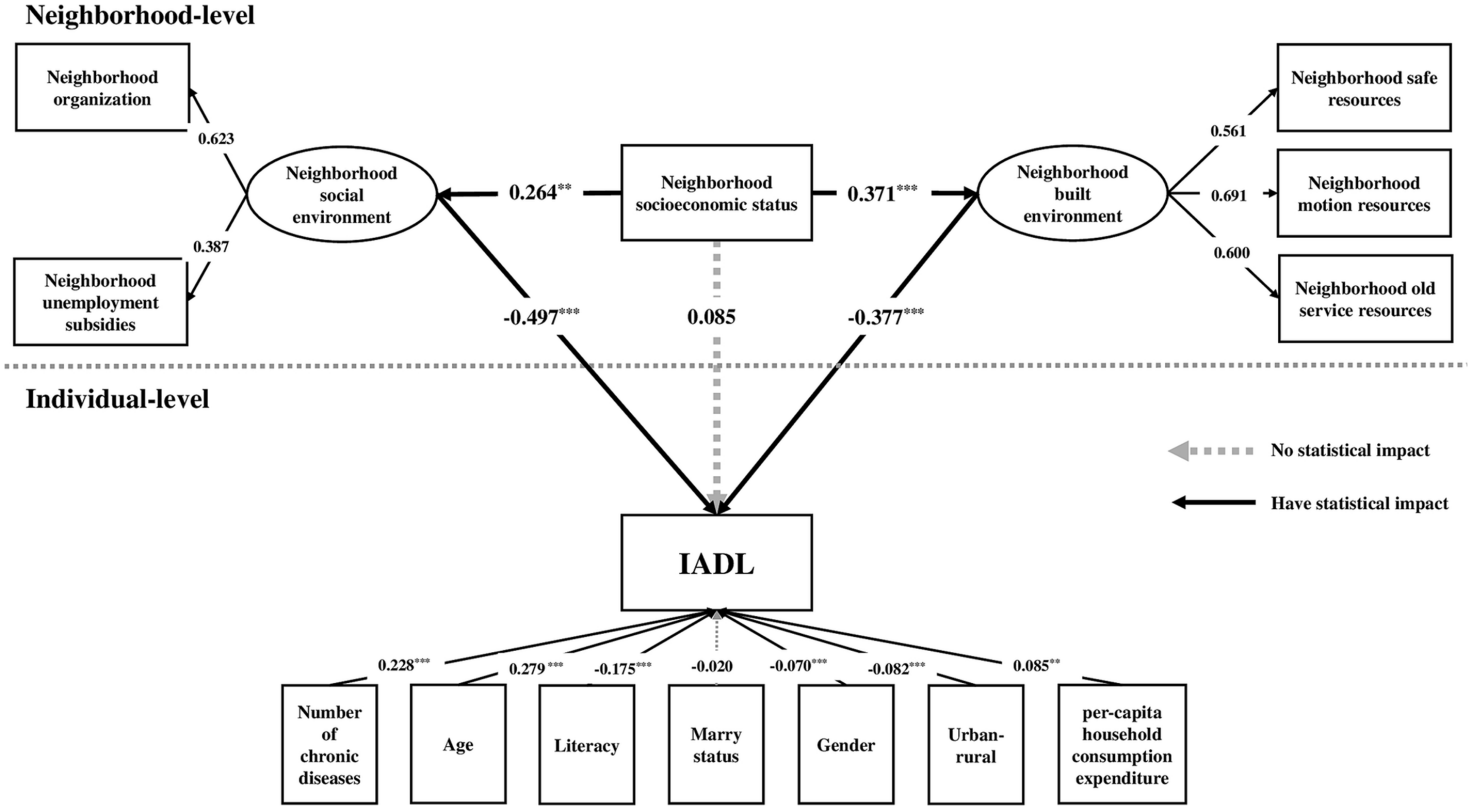
The multilevel structural model of neighborhood socioeconomic status, neighborhood environment and ADL. ^***^p < 0.01, ^**^p < 0.05.

Based on the measurement model, the dependent variable, independent variables, and seven control variables were entered into the multilevel structural model, and the model was shown in Figure 2. The estimates of the model fit index were as follows: χ^2^/df = 1.02*, p* = 0.42, RMSEA = 0.002, CFI = 0.998, TLI = 0.994, SRMR-W = 0.000, SRMR-B = 0.188, which indicated a good model fit.

The multilevel structural model showed that both neighborhood built environment and social environment were significantly associated with neighborhood socioeconomic status (Figure 2). The standardized coefficients were 0.387 (*p* < 0.001) and 0.221 (*p* < 0.05) respectively, indicating that the higher the neighborhood socioeconomic status, the better the neighborhood built environment and social environment. Moreover, neighborhood built environment (standardized coefficient = −0.284, *p* < 0.001) and neighborhood social environment (standardized coefficient = −0.412, *p* < 0.001) all had a statistical effect on ADL, which means that the risk of ADL loss decreased as respondents lived in better built environment and social environment in their neighborhoods. Moreover, the indirect effect standardized coefficient of neighborhood built environment was −0.110 (*p* < 0.05); the indirect effect standardized coefficient of neighborhood social environment was −0.091 (*p* < 0.05), and the deviation between them had no statistical significance (standardized coefficient = −0.019, *p* > 0.05), which meant that both neighborhood built environment and neighborhood social environment played an important mediating role. The standardized coefficient of the total effect of this model was −0.142 (*p* < 0.05), while the standardized coefficient of the direct effect of neighborhood SES on ADL had no statistical significance (standardized coefficient = 0.059, *p* > 0.05), meaning that neighborhood SES could affect on ADL indirectly through neighborhood built environment and neighborhood social environment rather than directly.

##### 3.3.1.2 Result of multilevel structural equation model on IADL

Based on the measurement model, the dependent variable, independent variables, and seven control variables were entered into the multilevel structural model, and the model was shown in Figure 2. The estimates of the model fit index were as follows: χ^2^/df = 1.03*, p* = 0.42, RMSEA = 0.002, CFI = 0.997, TLI = 0.993, SRMR-W = 0.000, SRMR-B = 0.188, which indicated a good model fit.

**Figure 3:**
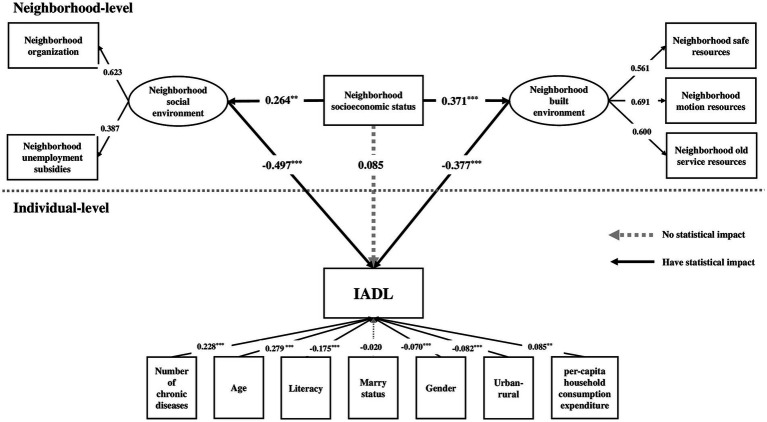
The multilevel structural model of neighborhood socioeconomic status, neighborhood environment and IADL. ^***^p < 0.01, ^**^p < 0.05.

The multilevel structural model showed that both neighborhood built environment and social environment were significantly associated with neighborhood socioeconomic status (Figure 3). The standardized coefficients for the neighborhood built environment and social environment were 0.371 (*p* < 0.001) and 0.264 (*p* < 0.05) respectively, indicating that the higher the neighborhood socioeconomic status, the better the neighborhood built environment and social environment. Furthermore, neighborhood built environment (standardized coefficient = −0.377, *p* < 0.001) and neighborhood social environment (standardized coefficient = −0.497, *p* < 0.001) all had a statistical effect on IADL, which means that the risk of IADL loss decreased as respondents lived in better built environment and social environment in their communities. Moreover, the indirect effect standardized coefficient of neighborhood built environment was −0.082 (*p* < 0.05); the indirect effect standardized coefficient of neighborhood social environment was −0.077 (*p* < 0.05), and the deviation between them had no statistical significance (standardized coefficient = −0.005, *p* > 0.05), which meant that both neighborhood built environment and neighborhood social environment played an important mediating role. The standardized coefficient of the total effect of this model was −0.109 (*p* < 0.05), while the standardized coefficient of the direct effect of neighborhood SES on IADL had no statistical significance (standardized coefficient = 0.050, *p* > 0.05), meaning that neighborhood SES could affect on IADL indirectly through neighborhood built environment and neighborhood social environment rather than directly.

## 4 Discussion

Using data from CHARLS and the multilevel structural equation model, this study identified a positive correlation and innovative mechanism between neighborhood SES and ADL/IADL loss among older adults, which was fully mediated by the neighborhood built environment and social environment. Neighborhoods with high SES were likely to have better neighborhood built environments and social environments, leading to a reduced likelihood of ADL/IADL losses among older adults.

This research indicated that neighborhoods with higher neighborhood SES tend to have superior built environments such as neighborhood safe resources, motion resources, and service resources for older adults, which aligns with Koohsari’s research (43). Koohsari’s study also found that these areas benefited from superior road conditions, including higher densities of streets and superior-quality road surfaces. Additionally, these neighborhoods boasted cleaner and safer environments and had a lower likelihood of being exposed to violence and disorder (21). As an important part of community construction, community sports facilities play an important role in increasing physical activity and participation among community residents. Studies have found that moderate-to-high levels of physical activity are protective factors for ADL in older adults (44). The more available neighborhood motion resources there are, the higher the physical activity levels of older adults, which contributes to the improvement of physical function and effectively prevents the onset or worsening of ADL in older adults (45), as well as being a prerequisite for them to achieve IADLs. In addition, communities with more safety resources and a higher levels of security are more likely to encourage older adults to engage in neighborhood activities, which is particularly relevant for their IADLs. Research indicates that neighborhood insecurity can increase negative emotions and feelings of unease among older adults. This, in turn, can limit their ability to travel and partake in physical activity, which can limit their capacity for IADL and potentially increase the incidence of ADL (33). Abundant neighborhood living resources, such as grocery stores, farmers’ markets and supermarkets, make life more comfortable for older adults. This allows them to maintain an independent lifestyle, ultimately improving their ability to manage money, go shopping, thus slowing down the aging process. Neighborhood service resources for older adults, such as activity centers, can enrich their lives, expand their social scope, strengthen neighborhood cohesion, effectively increase the frequency of older adults’ activities, and thereby benefit the IADL, likely reducing the incidence of ADL loss.

The neighborhood social environment also acted as a complete mediator in the interaction between neighborhood SES and ADL/IADL in older adults. This study indicated that neighborhoods with higher neighborhood SES tend to have better social environments such as neighborhood unemployment subsidies and neighborhood organizations, which is consistent with present research (46, 47). The existence of various social organizations in the community, such as calligraphy and painting associations, dance teams, and older adults’ associations, not only helps to cultivate the interests of the older adults, but also improves the frequency of their physical activity and social interaction. At the same time, it can create a good community atmosphere, enhance the sense of neighborhood belonging and cohesion of residents, and thus maintain their overall health. Studies have shown that communities with higher levels of neighborhood trust and social capital have higher rates of socialization, healthier lifestyles, and lower rates of ADL/IADL loss among older adults (7, 31). Drageset et al. (48) found that participation in neighborhood organizations significantly reduced the likelihood of ADL loss among older adults compared to those who did not participate. Neighborhood economic security policies, such as unemployment benefits, can facilitate community-based care for older adults and provide some financial assistance to unemployed community residents to ensure basic livelihoods and access to health care. In addition, financial allowances provide an economic basis for their participation in community activities. These can be effective in reducing the likelihood of ADL/IADL loss and adverse health outcomes among them due to economic factors (49).

Additionally, when assessing the neighborhood SES, it was found that the neighborhood per-capita net income was a more sensitive indicator than the neighborhood literacy index. One possible explanation is that neighborhood SES may be more closely linked to material resources of the neighborhood in daily life. Inequality in neighborhood per-capita net income, as an indicator of neighborhood SES, tends to lead to an uneven distribution of resources across communities, resulting in differences in the built and social environments of communities. In addition, since the neighborhood literacy index was a synthetic indicator that explained only 68% of the raw information, it may not accurately reflect the relationship between community education and ADL/IADL.

## 5 Limitations

Firstly, this cross-sectional study cannot confirm the causal relationship between neighborhood socioeconomic status and ADL/IADL in older adults. Besides, the community-level data did not encompass supplementary details such as occupational category, thus decreasing the comprehensiveness of the neighborhood SES measurement. Additionally, as CHARLS 2011 did not examine the task of making phone calls, our research on IADL only encompassed five components, which may make the status of IADL loss in older adults underestimated. Furthermore, the database only compiled neighborhood data in 2011, limiting the findings of this study to the association between SES and ADL/IADL in the older adult population during that time. Further research with updated data is necessary to reflect the present situation.

## 6 Conclusion

This study found an important process by which neighborhood SES has an indirect impact on ADL/IADL through the neighborhood’s built and social environment. Therefore, this study highlights the need to address ADL/IADL loss in older adults residing in neighborhoods with low socioeconomic status, and advocates for intervening in the occurrence of ADL/IADL loss through feasible improvement of the neighborhood environment. This study offered an economically viable solution for community-based interventions to improve functional health in older adults, and provided valuable input for enhancing the current model of aging within the community and promoting active aging.

## Data availability statement

Publicly available datasets were analyzed in this study. This data can be found here: 2011 CHARLS Wave1 (Baseline), https://charls.charlsdata.com/pages/Data/2011-charls-wave1/zh-cn.html.

## Ethics statement

The studies involving humans were approved by Peking University Institutional Review Board (PU IRB). The studies were conducted in accordance with the local legislation and institutional requirements. The participants provided their written informed consent to participate in this study.

## Author contributions

XT and XR contributed to conceptualization, visualization, and writing—review and editing. XT contributed to writing—original draft. HZ contributed to data curation, methodology, and formal analysis. XR contributed to supervision and validation. All authors contributed to the article and approved the submitted version.
